# Yield, Physiological Performance, and Phytochemistry of Basil (*Ocimum basilicum* L.) under Temperature Stress and Elevated CO_2_ Concentrations

**DOI:** 10.3390/plants10061072

**Published:** 2021-05-27

**Authors:** T. Casey Barickman, Omolayo J. Olorunwa, Akanksha Sehgal, C. Hunt Walne, K. Raja Reddy, Wei Gao

**Affiliations:** 1 North Mississippi Research and Extension Center, Mississippi State University, Verona, MS 38879, USA; ojo26@msstate.edu; 2 Department of Plant and Soil Sciences, Mississippi State University, Mississippi State, MS 39762, USA; as5002@msstate.edu (A.S.); chw148@msstate.edu (C.H.W.); krreddy@pss.msstate.edu (K.R.R.); 3 USDA UVB Monitoring and Research Program, Natural Resource Ecology Laboratory, Department of Ecosystem Science and Sustainability, Colorado State University, Fort Collins, CO 80523, USA; wei.gao@colostate.edu

**Keywords:** Genovese cultivar, photosynthesis, stomatal conductance, chlorophyll, carotenoids, antioxidant defense metabolites

## Abstract

Early season sowing is one of the methods for avoiding yield loss for basil due to high temperatures. However, basil could be exposed to sub-optimal temperatures by planting it earlier in the season. Thus, an experiment was conducted that examines how temperature changes and carbon dioxide (CO_2_) levels affect basil growth, development, and phytonutrient concentrations in a controlled environment. The experiment simulated temperature stress, low (20/12 °C), and high (38/30 °C), under ambient (420 ppm) and elevated (720 ppm) CO_2_ concentrations. Low-temperature stress prompted the rapid closure of stomata resulting in a 21% decline in net photosynthesis. Chlorophylls and carotenoids decreased when elevated CO_2_ interacted with low-temperature stress. Basil exhibited an increase in stomatal conductance, intercellular CO_2_ concentration, apparent quantum yield, maximum photosystem II efficiency, and maximum net photosynthesis rate when subjected to high-temperature stress. Under elevated CO_2_, increasing the growth temperature from 30/22 °C to 38/30 °C markedly increased the antioxidants content of basil. Taken together, the evidence from this research recommends that varying the growth temperature of basil plants can significantly affect the growth and development rates compared to increasing the CO_2_ concentrations, which mitigates the adverse effects of temperature stress.

## 1. Introduction

Climate change remains an important challenge affecting the attainment of global food security as it negatively impacts the growth and development of crops. Several studies have demonstrated higher atmospheric carbon dioxide (CO_2_) concentrations, extreme temperature conditions, and other extreme weather events as evidence of climate change [1,2]. Global atmospheric CO_2_ is rising (above 415 ppm in 2020). It is projected by climate models to reach the range of 540 to 970 ppm by 2100 because of human activities, declining carbon sinks, and natural global cycles [3,4]. Recent climate models have also predicted that global air temperature may experience increments in the range of 1.5 and 4.5 °C in the next century due to the increasing levels of atmospheric CO_2_ and other greenhouse gases at an alarming rate [1,5]. Atmospheric CO_2_ and temperature are critical in the photosynthesis, physiological, and developmental processes that occur in many crops, especially C3 crops [6,7]. Thus, anticipated increasing atmospheric CO_2_ and temperature will affect several crops’ growth and development, including basil (*Ocimum basilicum*). Increased environmental fluctuations resulting from climate change have been forecasted in many agricultural regions [8], and this poses a bane to achieving sustainable food production. Hence, it is pertinent to understand the mechanisms associated with basil’s response to elevated atmospheric CO_2_ and temperature stress to managing future production.

Basil is an important herbaceous aromatic plant with a noteworthy contribution to enhancing cuisine nutrition, healthy living, and landscape aesthetics. Globally, a large proportion of high-quality basil is cultivated for its essential oil, dry leaves, and flowers [9,10]. Studies have revealed different curative properties of basil, such as lowering blood pressures and fevers, reducing glucose and cholesterol levels in the blood, suppressing muscle spasms and inflammation, and strengthening the body’s natural activity [9].

In general, basil is widely adapted and grown throughout the globe. However, it is most suitable for warmer temperatures [11,12]. The optimum temperature for basil growth is in the range of 25 and 30 °C, while the minimum temperature at which basil can survive is 10.9 °C [11,13]. Recent evidence suggests that temperature significantly affects the growth and development of basil plants. For example, Chang et al. [11] indicated that increasing the growth temperature of basil to 30 °C culminated in the maximum rate of net photosynthesis (P_n_), transpiration rate (*E*), and stomatal conductance (g_s_), with resultant benefits on basil yields. Though basil is considered heat-tolerant, a temperature above 38 °C has been noted to cause detrimental effects on the yield, especially during the reproductive stages of development [11,14]. Temperature stress perturbs plant metabolism due to its damaging impact on the binding affinity and structure of proteins and enzymes, thereby resulting in the build-up of undesirable toxic intermediates, disjoining of diverse reactions, and increased levels of reactive oxygen species (ROS) [15]. Thus, many farmers usually resort to growing basil plants early in the growing season to avoid harmful environmental stress factors such as heat and drought, which can cause the plants to produce elevated ROS in tissues. Elevated levels of ROS can have a devastating effect on the growth, development, and production of phytonutrients. However, early-season planting increases the risk of exposing basil crops to low-temperature stress conditions.

Basil is sensitive to low temperatures, mostly below 10 °C, resulting in damage to growth and developmental processes [16]. Chilling causes brown discoloration of interveinal leaf areas, increased leaf blade thickening, decreased plant growth, reduced postharvest shelf life, and deterioration of quality and marketability [11,16]. Many studies show that cold temperature stress can also have harmful effects on the physiological traits of basils. Kalisz et al. [17] demonstrated that cold temperatures have negative impacts on basil P_n_, *E*, and g_s_, which impaired plant growth and development. Additionally, the negative impact was identified to decrease photosystem II (PSII) activity (F_v_’/F_m_’) and variable-to-initial chlorophyll fluorescence (F_v_/F_o_) after a 16-day low-temperature treatment [17].

Moreover, basil leaves subjected to low-temperature stress were identified to experience chlorosis due to rapid degradation of carotenoid, chlorophyll, and antioxidant content [11,18]. It is important to note that the alteration of basil leaf pigments due to chilling stress could damage the photosynthetic apparatus, with detrimental impacts on plant growth and development. Previous studies have revealed that basil’s greenness is an indicator of chlorophyll content, usually used by consumers to determine its productivity [19]. Therefore, further research is required to quantify basil’s physiological response to low-temperature stress to promote the breeding of cold-tolerant genotypes of basil.

Several studies have indicated that increasing CO_2_ concentrations positively impact plant growth and development, primarily because of the significant role CO_2_ plays in respiration and photosynthesis [6,20]. Al Jaouni et al. [2] reported that biomass production increased by 40% along with the photosynthetic and respiratory rate of basil, which significantly improved by 80% when atmospheric CO_2_ was increased from 360 to 620 ppm. The improved photosynthetic rate was attributed to the role of elevated atmospheric CO_2_ in repressing the oxygenation reaction of Rubisco, leading to improved carbon gains [20]. Previous studies by Gillig et al. [21] also found that basil grown under 1500 ppm of CO_2_ had a significant increase in their chlorophyll concentrations due to the accumulation of large grains of starch and non-structural carbohydrates. The levels of antioxidant defense metabolites, such as fumarate, glutamine, glutathione (GSH), ascorbic acid (ASC), phylloquinone (vitamin K1), anthocyanins (Anth), and most flavonoids and minerals were significantly improved by elevated CO_2_ [2]. Hence, the beneficial impacts of elevated atmospheric CO_2_ can counteract the adverse impacts of low- and high-temperature stress on basil physiology and phytochemistry. Even more than that, the benefits of improved growth rate in basil due to elevated CO_2_ could facilitate harvesting early, which will aid more crop cycles each year and contribute positively to food security.

Studies have been conducted to find out the influence of elevated CO_2_ and temperature stress on basil’s growth and photosynthesis. However, there has been a lack of information on the interactive impacts of CO_2_ and temperature on basil plants’ physiological and phytochemical performance. Hence, this study aims to evaluate the interactive impacts of elevated CO_2_ and temperature stress on photosynthesis parameters, carotenoids, and chlorophylls content of basil plants. Moreover, we evaluated important biochemical parameters acting as enzymatic and nonenzymatic antioxidant defense metabolites responsible for cellular osmotic adjustments in stressed plants.

## 2. Results

### 2.1. Gas Exchange Parameters

The results showed that temperature and its interaction with CO_2_ significantly (*p* < 0.001) affected the P_n_ of the basil plant (Table 1). However, elevated CO_2_ had no significant effect (*p* > 0.05) on P_n_. Under ambient CO_2_, high-temperature stress increased P_n_ by 72%, while low-temperature stress decreased the P_n_ by 38%. Correspondingly, under elevated CO_2_, high-temperature stress increased the P_n_ of basil by 45%, while low-temperature stress decreased the P_n_ by 21%, compared to the control treatments.

Additionally, temperature treatments significantly impacted the g_s_ and *E* of basil plants (Table 1). Specifically, the g_s_ and *E* of the basil plant were significantly increased due to exposure to high-temperature stress. Under low-temperature stress, g_s_ and *E* were reduced by 63% and 72%, respectively. Although elevated CO_2_ had no significant effects (*p* > 0.05) on g_s_ and *E*, there was a decreasing trend of g_s_ and *E* at elevated CO_2_ (Table 1). In contrast, the C_i_ concentrations of basil plants were significantly affected by both temperature and CO_2_ (*p* < 0.001), with significantly higher C_i_ at elevated CO_2_ as compared to the ambient CO_2_.

The C_i_/C_a_ was significantly different from temperature and CO_2_ stresses, but there was no difference from the control treatment when CO_2_ interacted with temperature stress. The photosynthetic ETR of basil reduced by 22% when exposed to low-temperature treatment and increased by 10% under high-temperature treatment compared to the control treatment. Similarly, the quantum efficiency (F_v_′/F_m_′) was affected by temperature stress and its interaction with CO_2_ (Table 1). The F_v_′/F_m_′ was decreased at low-temperature stress at ambient CO_2_, whereas F_v_′/F_m_′ increased when interacted with elevated CO_2_ at both low- and high-temperature stresses.

### 2.2. Chlorophyll Content and Total Carotenoids

**Figure 1:**
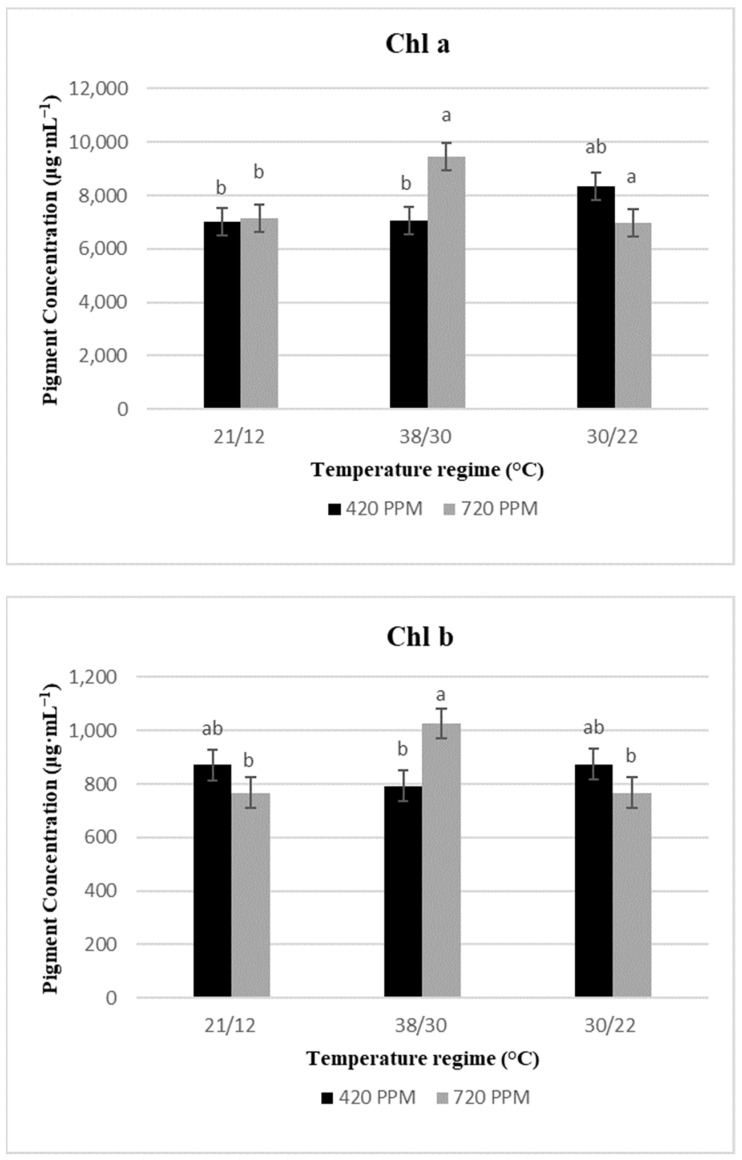

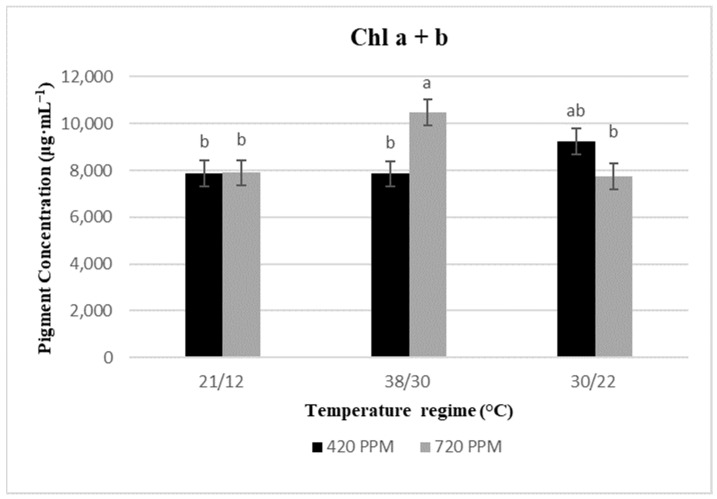
Chlorophyll a (Chl a), and chlorophyll b (Chl b), and total chlorophyll (Chl a + b), concentrations of basil plants under no temperature stress (Control), low-temperature stress, and high-temperature stress at 420 and 720 ppm of CO_2_ concentration. The standard error mean was Chl a = 506.33, Chl b = 57.19, and Chl a + b = 547.56. Different low case letters indicate significant difference at *p* = < 0.05 by least significant difference.

Compared to the control treatments, low-temperature stress when interacted with elevated CO_2_ caused a loss of pigment content in basil leaves by decreasing Chl a, b, and total chlorophyll (a + b) content by 1%, 12%, and 2%, respectively (Figure 1). While high-temperature stress at elevated CO_2_ significantly increased Chl a, b, and total chlorophyll (a + b) by 35%, 18%, and 33%, respectively. However, temperature stress only had significant effects (*p* > 0.001) on the total xanthophyll of the basil plant (Table 2). At ambient CO_2_, the total xanthophyll content was higher under heat stress, whereas it was lower under cold stress than in the control treatments. Compared to the control treatment, the carotenoid cycle (violaxanthin (V) + antheraxanthin (A) + zeaxanthin (Z)) decreased significantly under both low- and high -temperature stresses. Analogous results were observed for ZA/ZAV at elevated CO_2_. Elevated CO_2_ and its interaction with temperature stress significantly decreased the proportion of both lutein and neoxanthin when compared to the control treatments. However, temperature stress and its interaction with CO_2_ had no significant (*p* < 0.05) effects on the β-carotene. β-carotene was atypically much lower (24%) under elevated CO_2_ than in the ambient CO_2_ in the present study. The concentration of violaxanthin revealed no effects with temperature or elevated CO_2_ treatments.

### 2.3. Total Phenolics

**Figure 2:**
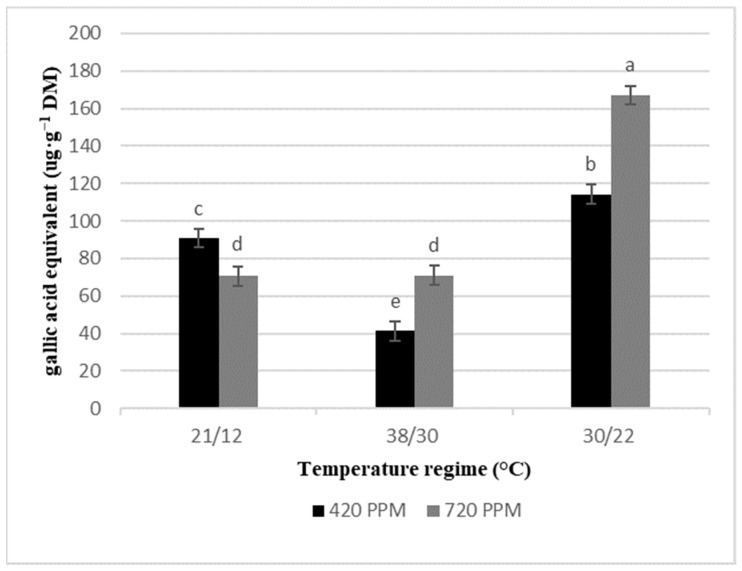
Total phenolic of basil leaf tissue subjected to no temperature stress (Control), low-temperature stress, and high-temperature stress at 420 and 720 ppm of CO_2_ concentration. Total phenolic content is presented as gallic acid equivalent concentration ug·g^−1^ dry mass (DM). The standard error mean was total phenolic = 5.133. Different low case letters indicate significant difference at *p* = < 0.05 by least significant differences.

Conflicting with the pattern of changes of chlorophyll contents, total phenolics reduced (7%) in basil plants subjected to the interactions of high-temperature stress and elevated CO_2_ (Figure 2), whereas a significant increase of 10% was observed under low temperature at elevated CO_2_.

### 2.4. Epiticular Wax

**Figure 3:**
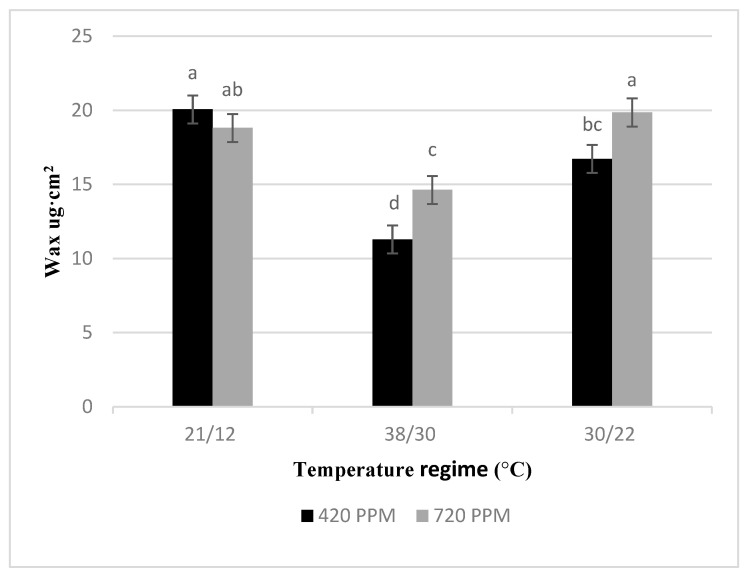
Average epicuticular wax content for basil plants grown without temperature stress (Control), with low-temperature stress and high-temperature stress at 420 and 720 ppm of CO_2_ concentration after 34 days of treatment. The standard error mean for wax was 0.9463. Different low case letters indicate a significant difference at *p* = < 0.05 by the least significant difference.

The basil plants showed a significant reduction in leaf wax content when subjected to both low- and high-temperature stresses and elevated CO_2_ (Figure 3). However, there was no interaction effect between CO_2_ and temperature treatments on basil leaf wax content.

### 2.5. Antioxidant and Oxidative Parameters

Interactions between temperature and CO_2_ significantly affected the content of MDA and GSH only (Table 3). Basil grown at low temperature under elevated CO_2_ significantly increased the MDA content by 150%, whereas the MDA content decreased by 43% under elevated CO_2_ at high temperature. In contrast, the total GSH levels of basil were markedly increased by 43% under high-temperature stress at elevated CO_2._ It decreased by 2% when subjected to low-temperature treatment at elevated CO_2_ related to the control. There was only a small difference between the GSH content under low-temperature stress at ambient CO_2_ and low-temperature stress at elevated CO_2_. Compared to the control treatments, the SOD content increased significantly under elevated CO_2_ at low and high-temperature stresses. Additionally, elevated CO_2_ alone was discovered to increase the ASC and TRE content of basil substantially by 89% and 41%, respectively, compared to the control treatments. However, temperature and elevated CO_2_ treatments and their interactions had no significant effects on H_2_O_2_ content.

## 3. Discussion

Abiotic stress as a result of increased fluctuations of temperature and atmospheric CO_2_ affects the productivity of many important crops, including basil [17,22]. In particular, extreme temperature stress during seedling growth reduced the yield of basil. Early season sowing is one of the methods for avoiding yield loss in basil due to high temperatures. However, when producers plant basil earlier in the season, the crop can be exposed to sub-optimal temperatures. Previous research has indicated that elevated CO_2_ is beneficial to C3 crop growth and development [6,23]. The connection to whether increasing CO_2_ from the ambient concentrations is beneficial in mitigating the adverse impacts of temperature stress in basil is yet to be determined. Hence, we evaluated the physiological and phytochemistry of basil subjected to varying temperature stress and CO_2_ concentrations.

The current research indicated that P_n_ and g_s_ reduced with low-temperature stress and increased when subjected to high-temperature stress. These observations agree with the results of Ribeiro et al. [16], Kalisz et al. [17], and Balasooriya et al. [24], who revealed a negative impact of chilling temperatures on basil photosynthesis. Basil plants lower the g_s_ of their leaves to adapt adequately to low-temperature stress. The lower g_s_ are linked to preventing leaf water loss (wilting), which are directly related to decreases in P_n_ and C_i_ concentration. However, in the current study, there were no observations of wilting plants due to decreased g_s_. The lower g_s_ and *E* and decreased P_n_ and C_i_ further complements the results of Saibo et al. [25], who proposed that reduced g_s_ are an essential factor influencing P_n_ decrease compared to non-stomatal limitations including reduced -Rubisco activity and -energy consumption. However, C_i_/C_a_ was observed to reduce under low temperature, suggesting that P_n_ reduction could also be due to decreased C_i_/C_a_. The accelerated chlorophyll pigment degradation of basil plants observed when elevated CO_2_ interacted with low-temperature stress could also be responsible for decreased P_n_. The results of this current study match those observed in basil by Gillig et al. [21]. Basil plants subjected to low-temperature stress exhibit lower gas exchange, decreased g_s_, and *E*, thereby explaining a decrease in the metabolic synthesis of metabolites, cell wall formation, and overall growth. Conversely, when basil plants are subjected to high-temperature stress, there is an increase in metabolic processes, gas exchanges, and morphological changes such as plant height. It is important to remember that fluctuations in temperature have a dramatic effect on plants’ metabolic functions that can lead to morphological and biochemical changes that can ultimately decrease crop yields.

Importantly, the adverse effects of the chilling temperature on P_n_ were ameliorated when interacted with elevated CO_2_. The increased CO_2_ inside the leaf would increase leaf photosynthesis through increased activity of the rubisco enzyme and reduced photorespiration [6,24]. In contrast, the high-temperature stress was observed to increase the g_s_, *E*, C_i_, apparent quantum yield, maximum photosystem II efficiency, and finally, maximum P_n_ of basil plants. This observation could be linked to the positive impacts of high-temperature treatments detected on the morphological parameters of basil plants [26]. Comparable results were accounted for by Chang et al. [11]. However, elevated CO_2_ did not have significant effects on g_s_ of basil plants in this study. Hence, proposing that the interactive impacts of elevated CO_2_ and temperature stress on P_n_ were not linked with changes in g_s_ under temperature stress because reduced g_s_ were already instigated by low-temperature stress. Photosynthetic ETR and F_v_′/F_m_′ also demonstrated a similar pattern with P_n_ and g_s_. The results additionally propose that the raised P_n_ and g_s_ under high-temperature stress could be responsible for the increased ETR chain under temperature stress. F_v_′/F_m_′ is sensitive to chilling conditions and therefore decreased F_v_′/F_m_′ can show stress via photo-inhibition. Moreover, the occurrence of photoinhibition in basil due to chilling stress could result to increased production of ROS in basil, which causes oxidative damage in plants. Kalisz et al. [17] attributed the F_v_′/F_m_′ reduction to the presence of photochemically inactive reaction centers and reduced ETR, which was also observed in this present study. On the contrary, elevated CO_2_ interacted with temperature stress to remarkably attenuated the damage caused by chilling stress on P_n_ and promoted F_v_′/F_m_′ by maintaining proper redox balance. Generally, photosynthesis is perhaps the most susceptible parameter to physiological processes, especially when C3 (basil) plants are subjected to low-temperature stress [27]. Thus, when basil plants are exposed to chilling stress, a decrease of P_n_, g_s_, *E*, and metabolites such as chlorophylls and carotenoids can have a considerable impact on plant growth and development.

Similar to most of our results, increased growth temperature from 30/22 °C to 38/30 °C under elevated CO_2_ produced more chlorophyll content of basil plants. Specifically, Chl a and Chl b increased 18% and 35%, respectively. These results suggest that subjecting basil to heat stress and elevated CO_2_ does not cause a drastic loss in Chl content, and this matches those observed in thermophilic plants [28,29]. Zhou et al. [30] reported that in tomato plants, a significant increase of 35% and 31% in Chl a and Chl b was observed, respectively, when the growth temperature increased from 25/20 °C to 38/30 °C. On the other hand, cold temperature stress under elevated CO_2_ significantly decreased the concentrations of basil Chl a with no change in the concentration of Chl b. Corroborating the present results, Kalisz et al. [17] and Liu et al. [31] reported a significant reduction in cold-sensitive crops when subjected to chilling stress. Contrary to previous research, it can be conceivably hypothesized that elevated CO_2_ does not mitigate the adverse effects of chilling stress on basil chlorophyll concentrations. The data also suggests that reduction in chlorophyll under low-temperature stress may be due to decreased metabolic functions that induce growth and development and production of metabolites to protect the plants. Conversely, under high-temperature stress conditions, growth and metabolic processes increase due to elevated P_n_, g_s_, and *E*.

Temperature is an important factor that regulates several compounds in basil that have health benefits to humans, including carotenoids, chlorophylls, phenolics, and epicuticular wax [10]. Previous research by Al-Huqail et al. [18] found that basil under any stress conditions caused a decrease in its carotenoid content. For instance, both cold and heat stress impair the xanthophyll cycle pigments of basil plants in the current study. Likewise, elevated CO_2_ caused a decrease in the xanthophyll cycle of basil plants compared to control treatments. Loladze et al. [32] ascribed the negative impacts of CO_2_ levels on the xanthophyll cycle of C3 plants to reduce non-photochemical quenching (NPQ) integral photoprotection. Thus, these results may be due to more of the energy produced by increasing CO_2_ levels is directed toward photosynthesis and less toward heat dissipation. However, subjecting basil plants to low-temperature stress led to a significant increase in total phenolics content. While under high-temperature treatment, total phenolics of basil decreased considerably. These findings contradict many studies [18,33,34] because increased phenolics are usually associated with thermophilic plant defense’s mechanism against heat stress. Moreover, epicuticular wax decreased significantly both under heat and cold stress conditions, which is unexpected because increased wax content is always utilized as a physiological trait for selecting thermophilic plants [35].

Furthermore, the decreased Chl, P_n,_ g_s_, *E*, and F_v_′/F_m_′ observed in this study could promote a significant proportion of the light energy utilized during photosynthesis to induce excessive accumulation of ROS in basil tissues, thereby impairing the overall growth of the plant. Previous studies have shown that temperature stress increases ROS production (e.g., H_2_O_2_ and O_2_), which causes oxidative stress in plants [36]. However, temperature and elevated CO_2_ treatments and their interactions had no significant effects on H_2_O_2_ content in this study. Thus, suggesting that heat stress did not induce oxidative damage in basil plants, which could further support basil’s tolerance to heat stress. Moreover, Al Jaouni et al. [2] demonstrated that elevated CO_2_ positively impacts basil antioxidant compounds. Correspondingly, results from this study indicated that increasing the CO_2_ concentration from 420 ppm to 720 ppm ameliorated the adverse effects of ROS by increasing TRE and ASC content. An increment in antioxidant enzyme activity has been described to be linked with plant resilience to heat stress [37]. It is important to note that the maximum increase was found in SOD content among the antioxidant enzymes, indicating that when basil was subjected to elevated CO_2_, this enzyme had played a critical part in decreasing the damaging consequences of ROS. SOD is considered an enzymatic antioxidant curbing the harmful effects of elevated ROS by catalyzing the dismutation of superoxide radicals to H_2_O_2_ and O_2_ [36]. Hence, the increased contents of SOD in basil plants under elevated CO_2_ would reduce the toxic effects of elevated ROS levels. Additionally, GSH is a nonenzymatic antioxidant, which minimizes the damage caused by ROS in plants and protects the photosynthetic apparatus from oxidative damage [27]. Hence, basil’s tolerance to heat stress could be linked to the increased GSH antioxidant levels observed in this study when high-temperature stress interreacted with elevated CO_2_.

Low-temperature stress frequently induces harm to cell membranes. MDA is a crucial indicator of membrane system injuries and cellular metabolism deterioration [31]. Basil grown at low temperature under elevated CO_2_ significantly increased the MDA content, whereas the MDA content decreased under high temperature at elevated CO_2_. This result suggests that the combination of low-temperature stress and elevated CO_2_ treatments is more damaging than the interaction of heat stress and elevated CO_2_. Thus, indicating increased resistance of basil to heat stress due to low levels of MDA observed.

## 4. Materials and Methods

### 4.1. Growth Condition and Plant Material

Basil ‘Genovese’ (Johnny’s Selected Seeds, Winslow, ME) seeds were planted in polyvinyl-chloride pots (15.2 cm diameter by 30.5 cm height). The lower part of each pot was loaded up with 500 g gravel while the upper parts were filled with a mixture of sand and soil (3:1 VV) in the soil-plant-atmosphere-research (SPAR) units at the Rodney Foil Plant Science research facility of Mississippi State University, Mississippi State, MS, USA, June-July 2019. The SPAR units can control environmental conditions, including temperature and CO_2_ concentration levels, at estimated set points. More information on the SPAR chamber subtleties were earlier portrayed by Reddy et al. [38] and Wijewardana et al. [39].

Six seeds previously selected by size and quality were planted in each pot, and approximately 14 days after sowing (DAS), the plants were thinned to one plant per pot. Throughout the experiment, basil plants were irrigated with full-strength Hoagland’s nutrient solution [40] three times daily (7 am, 12 pm, and 5 pm) via an automated computer-controlled drip system.

The experiment was organized in a randomized complete block design within a three by two factorial arrangement with temperature and CO_2_ treatments. A total of six SPAR chambers represented three blocks with ten replications. Each SPAR chamber consisted of three rows of pots with ten pots per row in each SPAR chamber. All environmental growing conditions, except for temperature and CO_2,_ were kept the same throughout the experiment.

### 4.2. Temperature and CO_2_ Treatments

Basil plants were randomly assigned to each chamber consisting of 20/12 (day/night), 30/22, and 38/30 °C in combination with ambient (420 ppm) or elevated (720 ppm) CO_2_ concentrations. The day- and night-time temperatures were respectively initiated at dawn and one hour after nightfall. Table 4 shows the average environmental conditions in which the experiment was conducted. During the period of this experiment, three temperature treatments, 20/12, 30/22, and 38/30 °C, were regarded as low, optimum, and high temperatures, respectively, for basil growth and development.

### 4.3. Physiology and Gas Exchange Measurements

The OJIP fluorescence readings were taken utilizing a FluorPen FP 100 (Photon Systems Instruments, Drasov, Czech Republic) on the second, most developed basil leaf. The minimal fluorescence (Fo), which was estimated at 50 μs when all PSII reaction centers are open, maximal fluorescence (Fm) when all PSII response focuses are shut, and the steady-state state fluorescence (Fs) were recorded in each plant at 17 DAT.

Analogous to the leaf chlorophyll readings, the photosynthesis and fluorescence parameters of basil leaf subjected to different treatments were recorded between 10 am and 12 pm with LI-6400XT portable photosynthesis system (LiCor Biosciences, Inc., Lincoln, NE, USA) at 17 DAT. These parameters include P_n_, *E*, g_s_, internal CO_2_ concentration (C_i_), electron transport rate (ETR), and the quantum efficiency (F_v_′/F_m_′). The internal to external CO_2_ ratio was calculated by the relationship C_i_/C_a_. The conditions of the leaf chamber were set at light intensity (PAR) of 1500 μmolm^−2^ s^−1^, the relative humidity of 50%, the CO_2_ concentration of 410 μmol mol^−1^, and the flow rate through the chamber was regulated to 500 mol s^−1^. The temperature of the chamber was set at the current temperatures (22, 30, or 38 °C) the readings were taken.

### 4.4. Carotenoid and Chlorophyll Analysis

Carotenoid and chlorophyll pigments were extracted and analyzed from freeze-dried basil tissues, according to Kopsell et al. [41,42], with few changes as portrayed in Barickman et al. [43].

### 4.5. Epicuticular Wax Content Determination

The epicuticular leaf waxes were extracted and quantitively analyzed in accordance with the method of Ebercon et al. [44] with minor modifications as described by Singh and Reddy [45].

### 4.6. Antioxidant and Oxidative Analysis

#### 4.6.1. Malondialdehyde (MDA)

The basil leaf samples were analyzed for MDA content by adapting the procedure used by Heath and Packer [46]. Fresh leaf tissue (500 mg) was homogenized in 0.1% trichloroacetic acid (TCA). The homogenate was centrifuged at 11,320× *g* for 5 min, and a 1mL aliquot of the supernatant was treated with 4 mL 0.5% thiobarbituric acid in 20% TCA; the mixture was heated at 958 °C for 30 min and afterward immediately cooled in an ice bath. After centrifugation at 5700× *g* for 10 min, the absorbance of the supernatant was recorded at 532 nm. The MDA content was determined by its extinction coefficient of 155 mM^−1^ cm^−1^ and expressed as nmol g^−1^ DW.

#### 4.6.2. Hydrogen Peroxide (H_2_O_2_)

The content of H_2_O_2_ was estimated according to the previously reported procedure of Mukherjee and Choudhuri [47]. Fresh leaf tissue (500 mg) was homogenized in 5 mL chilled acetone (80%) and filtered through Whatman filter paper, and 4 mL titanium reagent was added, followed by 5 mL ammonia solution. The mixture was centrifuged at 5030× *g*, and the supernatant was disposed of. The residue was dissolved with 1 M H_2_SO_4,_ and the absorbance was recorded at 410 nm. The extinction coefficient of H_2_O_2_ is 0.28 mmol^−1^ cm^−1^. The content of H_2_O_2_ in samples was acquired from a standard curve using pure H_2_O_2_ and expressed as µmol g^−1^ DW.

#### 4.6.3. Superoxide Dismutase (SOD)

The SOD activity was estimated using the method of Dhindsa et al. [48] with minor modifications reported by Awasthi et al. [49].

#### 4.6.4. Ascorbic Acid (ASC)

ASC was assessed following the method of Mukherjee and Choudhuri [47]. Details of extraction and analysis were described in Awasthi et al. [49].

#### 4.6.5. Glutathione (GSH)

Basil leaf samples were analyzed for reduced GSH according to the method of Griffith [50], with few changes detailed in Awasthi et al. [49].

#### 4.6.6. Trehalose (TRE)

Trehalose concentration was estimated according to the method of Trevelyan and Harrison [51] and the Anthrone method of Brin [52]. The enzymes associated with TRE metabolism were assayed as per the procedures of Pramanik and Imai [53], with few changes. Trehalose-6-phosphate synthase (TPS) activity was assayed, according to Hottiger et al. [54], which determined the release of UDP from UDP-glucose, involving glucose-6-phosphate. Trehalose-6-phosphate phosphatase (TPP) activity was assayed according to the method of Klutts et al. [55] by measuring the release of inorganic phosphate from trehalose-6-phosphate. Trehalase activity was determined by the activation of phosphorylation using cAMP (cyclic adenosine monophosphate) and assayed by measuring the glucose concentration [56].

## 5. Data Analysis

The experimental design was a randomized complete block in a factorial arrangement with three temperature treatments, two CO_2_ treatments, three-block, and ten replications. Data were analyzed using the PROC GLIMMIX analysis of variance (ANOVA) followed by mean separation. Statistical analysis of the data was performed using SAS (version 9.4; SAS Institute, Cary, NC, USA). The standard errors were based on the pooled error term from the ANOVA table. Duncan’s multiple range test (*p* ≤ 0.05) was used to differentiate between treatment classifications when F-values were significant for the main effects. Model-based values were reported rather than the unequal standard error from a data-based calculation because pooled errors reflected the statistical testing. Diagnostic tests were conducted to ensure that treatment variances were statistically equal before pooling.

## 6. Conclusions

The interaction of temperature stress and elevated CO_2_ significantly impacted the physiological processes and phytonutrient concentrations of basil plants. Decreasing the basil’s growth temperature to 20/12 °C significantly reduced P_n_ and g_s_, with detrimental impacts on the basil plants’ growth. Furthermore, low-temperature stress-induced excessive ROS production, which caused harm to the photosynthetic apparatus, as confirmed by reduced F_v_′/F_m_′, low ETR, and altered oxidized and reduced states of PSII and PSI. Additionally, the accelerated chlorophyll and carotenoid pigment degradation of basil plants were observed when elevated CO_2_ interacted with low-temperature stress.

Contrarily, elevated CO_2_ remarkably ameliorated the damage caused by low-temperature stress to photosynthetic apparatus. Thus, elevated CO_2_ promoted leaf C_i_/C_a_ and increased SOD, TRE, and ASC antioxidant levels. Basil, being a thermophilic plant, was observed to increase its chlorophyll and carotenoid concentration, apparent quantum yield, and maximum photosystem II efficiency when subjected to high-temperature stress. Likewise, increased growth temperature for basil under elevated CO_2_ produced more antioxidant content. The findings of this study recommend that varying the growth temperature of basil plants would significantly affect the growth and development rates of basil compared to increasing the CO_2_ concentrations, which mitigated the constraining effects of temperature stress.

## Figures and Tables

**Table 1:** Interactive effects of temperature stress and CO_2_ on net photosynthesis (P_n_), stomatal conductance to water vapor (g_s_), intercellular CO_2_ concentration (C_i_), electron transport rate (ETR), leaf transpiration rate (E), intercellular/ambient CO_2_ ratio (C_i_/C_a_), and the maximal quantum yield of photosystem II photochemistry (F_v_’/F_m_’), of basil plants. Measurements were taken on the fourth/fifth fully expanded leaf of plants grown without temperature stress (Control), with low-temperature stress, and high-temperature stress at 420 and 720 ppm of CO_2_ concentration between 37 and 38 days of treatment.

	P_n_	g_s_	C_i_	ETR	*E*	C_i_/C_a_^1^	F_v_^’^/F_m_^’^
Treatment	(μmol·m^2^·s^−1^)	(mol·m^2^·s^−1^)	(μmol·m^2^·s^−1^)	(μmol m^−2^·s^−1^)	(mmol·m^2^·s^−1^)		
420 PPM							
Control	24.48 c	0.38 b	295.09 d	187.33 ab	6.79 c	0.70 b	0.47 b
High Temperature	42.22 a	0.71 a	303.47 d	205.94 a	15.66 a	0.72 b	0.52 a
Low Temperature	15.20 d	0.14 c	237.64 e	146.46 bc	1.91 d	0.54 c	0.42 c
720 PPM							
Control	31.51 b	0.31 b	530.71 b	184.97 ab	6.67 c	0.74 b	0.51 a
High Temperature	35.51 b	0.63 a	597.80 a	183.19 abc	14.39 b	0.83 a	0.49 ab
Low Temperature	19.46 d	0.13 c	444.50 c	130.16 c	1.63 d	0.62 c	0.47 b
Treatment ^2,3^	***	***	***	*	***	***	**
CO_2_	NS	NS	***	NS	NS	**	NS
Trt *CO_2_	***	NS	*	NS	NS	NS	*

^1^ The measured intercellular CO_2_/ambient CO_2_ of LI-6400XT leaf cuvette. ^2^ SE— standard error of the mean; P_n_ = 1.5044; g_s_ = 0.03683; C_i_ = 15.4158; ETR = 17.876; *E* = 0.3881; C_i_/C_a_ = 0.02751; F_v_’/F_m_’ = 0.01425. ^3^ NS represents non-significant *p* > 0.05; *, **, *** represent significance levels at *p* ≤ 0.05, *p* ≤ 0.01, and *p* ≤ 0.001 respectively; within columns, values followed by the same letters are not significantly different.

**Table 2:** Interactive effects of temperature stress and CO_2_ on carotenoids concentration of basil leaf tissue. Leaf samples were taken from basil plants grown without temperature stress (control), with low-temperature stress and high-temperature stress at 420 and 720 ppm of CO_2_ concentration between 37 and 38 days of treatment.

Concentration (μg·g^−1^ Dry Mass)								
Treatment	Neo ^a^	Viol	Anth	Zea	Lut	β-car	Total Xan	ZA/ZAV ^b^
420 ppm								
Control	276.43 a	204.25 a	68.76 ab	163.94 a	793.09 a	509.66 a	436.95 a	0.53 a
High Temperature	252.16 abc	215.89 a	58.23 b	101.84 bc	710.64 ab	464.48 ab	375.97 bc	0.43 c
Low Temperature	264.59 ab	239.62 a	74.12 a	161.19 a	669.04 b	506.33 a	474.93 a	0.50 ab
720 ppm								
Control	222.36 c	208.03 a	74.29 a	157.75 a	561.09 c	384.95 bc	440.08 a	0.53 a
High Temperature	265.46 ab	226.79 a	43.14 c	78.38 c	687.31 b	390.65 bc	348.31 c	0.35 d
Low Temperature	235.20 bc	236.26 a	62.88 ab	119.79 b	552.75 c	342.73 c	418.93 ab	0.44 bc
Treatment ^c,d^	NS	NS	***	***	NS	NS	***	***
CO_2_	*	NS	NS	*	***	***	NS	*
Trt *CO_2_	*	NS	*	NS	*	NS	NS	NS

^a^ Neo—neoxanthin; Vio—violaxanthin; Anth—antheraxanthin; Zea—zeaxanthin; Lut—lutein; β-car—beta carotene; Xan—xanthophylls. ^b^ Xanthophyll cycle ratio = zeaxanthin to antheraxanthin/zeaxanthin to antheraxanthin to violaxanthin. ^c^ The standard error of mean was: Neo—14.10; Vio—14.55; Anth—7.17; Zea—11.27; Lut—35.85; Bcar—32.55; Total Xan—19.09; ZA/ZAV—0.025. ^d^ NS represents non-significant *p* > 0.05; *, *** represent significance levels at *p* ≤ 0.05 and *p* ≤ 0.001, respectively; within columns, values followed by the same letters are not significantly different.

**Table 3:** Interactive effects of temperature stress and CO_2_ on metabolites of basil leaf tissues. Leaf samples were taken from basil plants grown without temperature stress (Control), with low-temperature stress and high-temperature stress at 420 and 720 ppm of CO_2_ concentration between 37 and 38 days of treatment.

Concentration						
	nmol·g^−1^ DM	µmol·g^−1^ DM	Units/mg Protein	nmol·g^−1^ DM	µmol·g^−1^ DM	nmol·g^−1^ DM
Treatment	MDA ^a^	H_2_0_2_	SOD	ASC	TRE	GSH
420 ppm						
Control	0.0080 b	0.191 a	0.0304 c	0.101 b	0.0892 bc	0.192 b
High Temperature	0.0076 b	0.206 a	0.0306 c	0.123 ab	0.103 abc	0.188 b
Low Temperature	0.0074 b	0.198 a	0.0396 ab	0.106 b	0.0834 c	0.189 b
720 ppm						
Control	0.0078 b	0.183 a	0.0392 ab	0.191 a	0.126 ab	0.177 b
High Temperature	0.0046 b	0.206 a	0.0330 bc	0.150 ab	0.116 abc	0.275 a
Low Temperature	0.0200 a	0.266 a	0.0466 a	0.175 a	0.133 a	0.188 b
Treatment ^b,c^	**	NS	**	NS	NS	*
CO_2_	NS	NS	*	**	**	NS
Trt *CO_2_	**	NS	NS	NS	NS	*

^a^ MDA—malondialdehyde; H_2_0_2_—peroxide; SOD—superoxide dismutase; ASC—ascorbic acid; TRE—trehalose; GSH—glutathione. ^b^ The standard error of mean was MDA = 0.001976; H_2_0_2_ = 0.03045; SOD = 0.00272; ASC = 0.02317; TRE = 0.01383; GSH = 0.01776.^c^ NS represents non-significant *p* > 0.05; *, ** represent significance levels at *p* ≤ 0.05 and *p* ≤ 0.01 respectively; within columns, values followed by the same letters are not significantly different.

**Table 4:** Temperature stress treatments based on the percentage of daily evapotranspiration (ET) imposed at 14 days after sowing, mean day/night temperature, mean day chamber CO_2_ concentration, mean day/night vapor pressure deficit (VPD), and mean day/night evapotranspiration (ET) during the experimental period 38 days for each treatment.

Treatments		Measured Temperature (°C)	CO_2_ (ppm)	VPD (kPa)	Mean ET (H_2_O L·d^−1^)
		Day/night	Day	Day/night	Day/night
Control	30/22 °C, 420 ppm	26.27 ± 0.02	430.47 ± 0.98	1.82 ± 0.01	14.64 ± 1.41
Control + High CO_2_	30/22 °C, 720 ppm	26.34 ± 0.01	731.21 ± 1.52	1.98 ± 0.01	12.60 ± 1.27
High Temperature	38/30 °C, 420 ppm	32.16 ± 0.49	434.19 ± 1.21	2.80 ± 0.07	8.74 ± 0.64
Low Temperature	20/12 °C, 420 ppm	19.53 ± 0.56	431.08 ± 0.66	0.89 ± 0.08	8.59 ± 0.47
High Temperature + High CO_2_	38/30 °C, 720 ppm	32.09 ± 0.49	728.79 ± 0.83	2.87 ± 0.07	18.41 ± 1.86
Low Temperature + High CO_2_	20/12 °C, 720 ppm	19.56 ± 0.57	724.78 ± 0.35	0.95 ± 0.09	6.39 ± 0.37

## Data Availability

The data presented in this study are available on request from the corresponding author.
